# Ursolic acid rich *Ocimum sanctum* L leaf extract loaded nanostructured lipid carriers ameliorate adjuvant induced arthritis in rats by inhibition of COX-1, COX-2, TNF-α and IL-1: Pharmacological and docking studies

**DOI:** 10.1371/journal.pone.0193451

**Published:** 2018-03-20

**Authors:** Aftab Ahmad, Mohammed F. Abuzinadah, Huda M. Alkreathy, Babajan Banaganapalli, Mohd Mujeeb

**Affiliations:** 1 Health Information Technology Department, Jeddah Community College, King Abdulaziz University, Jeddah, Kingdom of Saudi Arabia; 2 Department of Medical Laboratory Technology, Faculty of Applied Medical Sciences, King Abdulaziz University, Jeddah, Kingdom of Saudi Arabia; 3 Department of Pharmacology, Faculty of Medicine, King Abdulaziz University, Jeddah, Kingdom of Saudi Arabia; 4 Princess Al-Jawhara Al-Brahim Center of Excellence in Research of Hereditary Disorders, King Abdulaziz University, Jeddah, Kingdom of Saudi Arabia; 5 Department of Genetic Medicine, Faculty of Medicine, King Abdulaziz University, Jeddah, Kingdom of Saudi Arabia; 6 Department of Pharmacognosy & Phytochemistry, School of Pharmaceutical Education and Research, Jamia Hamdard (Hamdard University), New Delhi, India; Indiana University, UNITED STATES

## Abstract

**Background:**

Ursolic acid (UA) is a promising molecule with anti-inflammatory, analgesic and potential anti-arthritic activity.

**Methods:**

This study was undertaken to make formulation and evaluation of *Ocimum sanctum* L. leaf extract (OLE) loaded nano-structured lipid carriers (OLE-NLCs) for improved transdermal delivery of UA. Different surfactants, solid lipids and liquid lipids were used for the preparation of NLCs. The NLCs were developed using emulsion solvent diffusion and evaporation method. Different physicochemical properties, entrapment efficacy, *in vitro* release evaluation, and *ex vivo* permeation studies of the prepared NLCs were carried out. The *in vivo* anti-arthritic activity of OLE-loaded NLC gel and control gel formulation (OLE free NLC gel) against Complete Freund's Adjuvant (CFA) induced arthritis in wister albino rats was also carried out.

**Results:**

OLE-NLCs were composed of spherical particles having a mean particle size of ~120 nm, polydispersity index of ~0.162 and zeta potential of ~ -27 mV. The high entrapment efficiency (EE) of UA ~89.56% was attained. The *in vitro* release study demonstrated a prolonged release of UA from the NLCs up to 12 h. The developed formulation was found to be significantly better with respect to the drug permeation amount with an enhancement ratio of 2.69 as compared with marketed formulation. The *in vivo* biological activity investigations, studies showed that the newly prepared NLCs formulation of OLE showed excellent anti-arthritic activity and the results were found at par with standard marketed diclofenac gel for its analgesic and anti-arthritic activities. These results were also supported by radiological analysis and molecular docking studies.

**Conclusion:**

The overall results proved that the prepared OLE-NLCs were very effective for the treatment of arthritis and the results were found at par with standard marketed the standard formulation of diclofenac gel.

## Introduction

Rheumatoid arthritis (RA) is a most common chronic inflammatory disease of an autoimmune origin. It affects a large population of the society, especially middle-aged people. Arthritis involves the inflammation of joints (affecting one or more joints), causing pain and stiffness in the joints. It causes destruction of cartilage, bone and affects synovial joints which lead to deformity, destruction and disability of joints. This is growing clinical health problem throughout the world. There are different types of arthritis like rheumatoid arthritis, gout, ankylosing spondylitis, psoriatic arthritis, septic arthritis, pseudo-gout, osteoarthritis, and juvenile idiopathic arthritis. The osteoarthritis is a degenerative joint disease and very commonly found among people. Arthritis might lead to poor physical function due to severe joint pain, hence it may adversely disturb the quality of life. The prevalence statistics of the arthritis reveal that Rheumatoid arthritis (RA) affects approximately 1% of the world population. Arthritis is widely prevalent in the American population and a leading cause of disability. According to the Centers for Disease Control and Prevention (CDC) data on arthritis in the United States; it has been reported that 54.4 million U.S. adults (approximately 25% of the total adult population) are suffering from arthritis. These figures of arthritis in adults are expected to increase up to 67 million by 2030. The total figures of doctor-diagnosed arthritis is projected to reach up to 78.4 million adults (25.9% of all adults) and the arthritis-attributable activity limitation in adults is expected to increase to 34.6 million (11.4% of all adults) by 2040 [1–2]. These data indicated that arthritis is a serious disorder which might be more prevalent than other known diseases like diabetes, cancer and AIDS. Therefore, there is an urgent need to address these growing problems of arthritis. Recent studies report that the occurrence of RA in Saudi Arabia is approximately found to be 2.2 per thousand people. Further, it is reported to be found more commonly in females in comparison to males, and prevalence of this disease increases with age. If RA patients remain untreated, it may lead to high morbidity and mortality. It is also said that RA has also resulted from cardiovascular diseases. In another recently conducted study in Saudi Arabia, the prevalence of hyperlipidemia in patients suffering from RA and its relationship with C-reactive protein level was investigated. It is reported that hyperlipidemia is frequently found among patients suffering from RA and significantly linked to the level of C-reactive protein (CRP) and disease activity. There are continuous efforts by the medical community and researchers to remove all obstacles in the management of RA around the world, particularly in Saudi Arabia [3–4]. The mechanism underlying the inflammatory progression of arthritis involved the infiltration of inflammatory cells into the joints, which causes the proliferation of synoviocytes and damage to the bone and cartilage. Certain pro-inflammatory cytokines e.g., TNF-α, interleukin-1 (IL-1), interleukin-1β (IL-1β), interleukin-6 (IL-6), interleukin-8 (IL-8), and interferon gamma (IFNγ) are considered as the primary mediators which have a vital role in the pathophysiology of RA. These pro-inflammatory cytokines promote inflammation by tissue destruction, angiogenesis, and leukocyte linkage in RA. Some new drugs have been used to target these primary mediators for the management of RA [5]. Although arthritis is considered as a disease of the joints, but abnormal immune responses may end up causing various articular manifestations. Furthermore, in certain cases, rheumatoid factor (RF) may also cause extra-articular findings. Various allopathic medications are employed in the management of arthritis. The corticosteroids (GCs) and non-steroidal anti-inflammatory drugs (Commonly referred as NSAIDs) are used as first line drugs of choice for the treatment of arthritis. NSAIDs are most frequently used drugs as compared with corticosteroids. The mechanism of action of NSAIDs involves the inhibition of cyclooxygenase enzymes both COX-1 and COX-2. GCs are steroid hormones, which act by binding to the glucocorticoid receptors. GCs plays vital role in the treatment of variety of diseases like asthma, autoimmune diseases, allergies, and sepsis etc. GCs have various pleiotropic actions with several potentially harmful side effects. Glucocorticoids are potent anti-inflammatory agents which can inhibit all stages of the inflammatory reactions. GCs are frequently used to treat a wide range of inflammatory diseases. Long term use of GCs use might lead to serious adverse effects like immune-suppression, fluid shifts, psychological changes and brain changes. Mechanism of corticosteroids action also involves the inhibition to the induction of COX enzyme. In addition to this, the corticosteroids also reduce the macrophage phagocytosis and interleukin-1 (IL-1) secretion in order to inhibit the release of lysosomal enzymes and collagenase [6]. The allopathic medications suffer from certain untoward effects. For instance sulfasalazine causes gastrointestinal disturbances, malaise and headache. Use of gold compounds is associated with skin rashes, mouth ulcers and blood dyscrasias and methotrexate may cause pulmonary fibrosis. Common side effects of NSAIDs include nausea, dyspepsia, vomiting, skin reactions [7]. Herbal medications on the other hand, are devoid of such untoward effects. Natural products in the form of the conventional dosage forms have been used for a long time. Use of nanotechnology in drug delivery technique is relatively new to pharmaceutical technology. The size range of nanoparticles is <250 nm. Because of their very small size they are benefitted with various properties that could be used in drug delivery techniques. Various nanoformulations have been prepared for herbal components and their rich extracts. They have been proven to provide additional benefit over the conventional dosage forms. For example, paclitaxel provides enhanced anti-tumor activity in the form of nano-particles as compared to its conventional dosage form. *Aloe vera* extract, *Ginkgo biloba* extract, curcumin and many more phytoconstituents have been modified into nano-type so as to provide additional benefit to the users, thereby increasing patient compliance [8–9].

**Fig 1:**
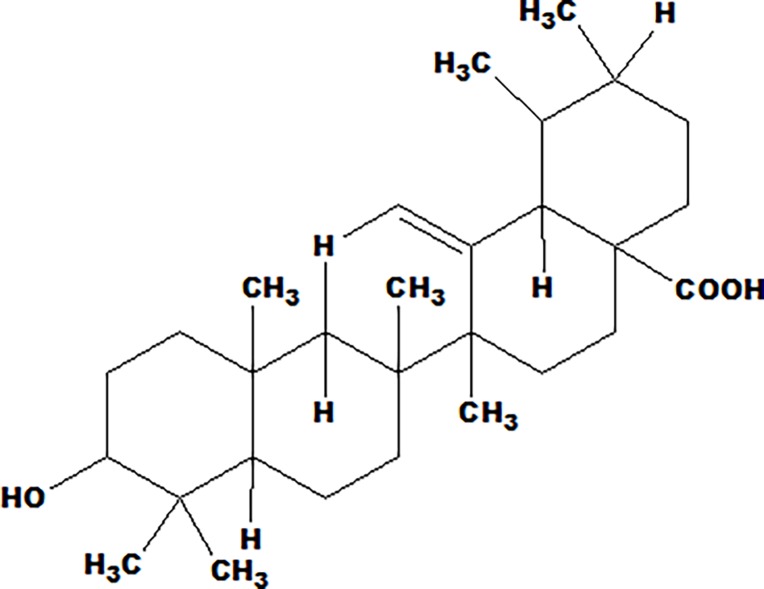
Ursolic acid (3β-hydroxy-urs- 12-en-28-oic acid).

In the present investigation a well-known Ayurvedic herbal drug *Ocimum sanctum* L was chosen for the undertaken research study. *Ocimum sanctum* is an important aromatic plant (Family Lamiaceae). It is commonly called as tulsi or holy basil. It is also known as *Ocimum tenuiflorum* and is frequently used by many in the treatment of variety of disorders/diseases throughout the world. It is inhabitant to the Indian subcontinent and is abundantly found in India. It is a very popular herb in Ayurvedic system of medicine and holds the highest regard for both its medicinal properties and religious beliefs. It is said to be an “elixir of life” [10]. Tulsi is known as “The Queen of Herbs,” and “Mother Medicine of Nature” [11–12]. In India, tulsi has been accepted in many religious customs and lifestyle practice for various health benefits. Daily use of tulsi is believed to promote general health, wellbeing and prevent various diseases and illness. In addition to this, several *in vitro* and *in vivo* scientific studies have been published throughout the world that provide the scientific basis of the medicinal properties of tulsi. Tulsi possess a wide range of pharmacological actions and used in the treatment for the variety of disorders/diseases like arthritis, asthma, anxiety, back pain, cardiac disorders, cough, dysentery, diarrhea, eye diseases, fever, genitourinary disorders, indigestion, gastric disorders, malaria, skin diseases, vomiting, ringworm, insect, scorpion, and snake bites. Tulsi is considered as a potent adaptogen which helps to improve the adaptation to stress and offer great support to maintain homeostasis [13–16]. The leaves have been reported to contain 0.7% volatile oil, comparing of eugenol (71%) and methyl eugenol (20%) and other constituents [17]. This is reported in the literature that leaves extract contains a higher concentration of ursolic acid [18]. Ursolic acid (UA) forms an indispensable part of Indian traditional system of medicine. Chemically speaking, UA is a triterpinoid and form an important constituent of Tulsi [19]. Moreover, it is also an important component of several herbal medicines marketed worldwide. UA (Fig 1) is known to possess analgesic, anti-inflammatory, anti-atherosclerotic, anti-cancer, anti-diabetic, anti-epileptic, hepato-protective, anti-hyperlipidemic, anti-fertility, anti-platelet aggregation, anti-tuberculosis and anti-HIV activities [20–25].

The preparation of crude extract is comparatively simple, easy, cheap and consume lesser time as compared with the manufacturing procedure of pure compounds. In addition to this, a crude extract contains natural active ingredients. The crude extracts also have potent pharmacological actions as compared to pure compound due to the synergistic outcome of the other compounds present in the extracts. Thus, in the current study, Ocimum leaf extract (OLE) was used instead of pure ursolic acid (UA). Nanostructured lipid carriers (NLCs) are lipid nano-particles of the 2^nd^ generation. These lipid nanoparticles were prepared after the first generation; solid lipid nanoparticles (SLNs) [26–27]. NLCs are distinct from SLNs with respect to their solid matrix composition. SLNs contains solid lipids only, on the other hand, NLCs consist of the mixture of liquid and solid lipids. NLCs exhibit more advantages as compared with than SLNs since NLCs present a smaller amount of lipid matrix which might offer extended loading capacity and NLCs also overcome a number of possible problems encountered with SLNs like expulsion of the drug for the period of the storage, lower drug loading and higher water content of SLNs distribution [28]. Further, the lipid nanoparticles the loaded active compounds have an additional advantage to prevent the degradation of the drug and also improve the penetration to the skin. The present study was aimed to develop herbal extract based NLCs to enhance bioavailability of UA and other constituents present in the extract for better management of arthritis with lesser adverse effects.

## Materials and methods

### Drugs and chemicals

Ursolic acid (UA), solid lipid Glyceryl Monostearate (GMS) and Complete Freund’s adjuvant (CFA) was procured from Sigma Chemical Company (MO, USA). Liquid lipid Capryol-90 was a kind offer from Gattefosse (Gattefosse, India). Surfactant Tween 80 was purchased from Thomas Baker (Chemicals) Ltd. (Mumbai, India). Mannitol, Carbopol-934 and High-performance Thin Layer Chromatography (HPTLC) grade methanol and water were purchased from S.D. Fine—Chemical Ltd. (Mumbai, India). The other chemical and reagents used for the study were of analytical grade and procured from an approved vendor.

### Preparation of Ocimum Leaf extract (OLE)

Ocimum Leaf extract (OLE) was prepared by supercritical carbon dioxide extraction (SFE) method. The dried powder of Ocimum leaf (200 gm) was extracted twice with SFE System (Prime Speed Applied Separation, USA) at 45°C temperature, 200 Psi pressure for 1.5 h. The obtained extracts were concentrated under decreased pressure in a rotary vacuum evaporator (HAHN SHIN, HS-2005 V-N) at 40°C. The extract obtained was stocked at low temperature and used for planned studies.

### Quantification of UA by High Performance of Thin Layer Chromatography (HPTLC)

The quantification of ursolic acid (UA) was performed using HPTLC (CAMAG, Switzerland). The samples of ursolic acid were smudged in the form of bands having width 5 mm with a CAMAG microliter (μL) syringe on pre-coated silica gel aluminum plate 60 F254 (20 cm ×10 cm with 0.2 mm thickness, E. Merck, Germany) using a Camag Linomat V (Switzerland) applicator. The mobile phase was composed of Toluene: Acetone: Formic acid (7.8:2.2:0.15 v/v/v). Densitometric scanning was carried out on CAMAG TLC scanner III in the absorbance mode at 245 nm.

### Preparation of Ocimum leaf extract loaded nanostructured lipid carriers (OLE- NLCs)

The solvent evaporation method was adopted to prepare the OLE-NLCs followed by ultrasonication [29–30]. GMS as solid lipid, capryol-90 as liquid lipid and OLE were dissolved in ethanol maintained in water bath at 80 ^0^C to form lipid phase. Ratio of total lipid and organic solvent was kept at 1:20. The aqueous phase was formulated by dispersing 3% Tween 80 in distilled water maintained at the similar temperature as that of lipid phase. The lipid phase was scattered in a hot surfactant aqueous solution pre-heated at the equal temperature added drop by drop to the aqueous phase and stirred continuously at 80 ^0^C until all the organic solvent was evaporated. The hot lipid nano-emulsion so obtained was a probe syndicated at 40% amplitude for 4 minutes. Lastly, the stirring of obtained nano-emulsion (O/W) was carried out on magnetic stirrer at 600 rpm for one hour approximately and then it was made to cool down at normal room temperature with continuous stirring. Finally the nano-emulsion was mixed with 3% mannitol. Blank NLC was also synthesized without the addition of OLE into the lipid phase.

### Evaluation of morphology, particle size and zeta potential

The dynamic light scattering (DLS) technique was used to measure the size and zeta potential by using Malvern Instruments (HAS- 3000, Malvern, UK). OLE-NLC suspension was mixed with the suitable medium and measurements of the particles were recorded in triplicate. The poly dispersity index (PDI) was established to determine the homogeneity [31]. The determination of the morphology of OLE-NLC was carried out by transmission electron microscopy (TEM Morgagni 268-D; FEI Company, Eindhoven, **N**etherlands). A single drop of the dispersion was placed to a carbon-coated copper grid and kept for one minute to let the OLE-NLC to stick to the carbon substrate. The residual dispersion was eliminated by absorbing the drop with a piece of filter paper. Subsequently, a drop of 1% phosphotungstic acid was kept on the grid and this sample was then allowed to dry in the air and then scanned. The image was taken by using Soft Q3 Imaging Viewer software.

### Entrapment efficiency

Entrapment efficiency (EE) was calculated in terms of UA content. The untrapped drug was removed by centrifugation of the sample at 4°C for 30 minutes at 14,000 rpm (REMI Cooling centrifuge, C-24, Mumbai) and the supernatant liquid was collected separately, then phosphate buffer was added to make dilution. The quantity of UA was estimated by HPTLC (CAMAG, Switzerland) method as illustrated above. The entrapment percentage of the UA was determined by using the equation given below [31].

$$EP=\frac{Ct-Cr}{Ct}X100$$

In the equation, Ct means is the concentration of total UA and Cr indicates the concentration of free UA.

### Preparation of Rat's skin for permeation studies

Wistar albino rats (200–250g) were used to take their skin for permeation studies of the NLCs. Care of the rats was taken in accordance with the institutional guidelines. The rats were scarified by cervical dislocation with utmost care to avoid pain to the animals. The skin was surgically removed safely from the abdominal area. The hairs were shaved and eliminated with help of an electric clipper and subcutaneous fat was eradicated by treating the excised skin with isopropyl alcohol (IPA). The excised skin was then thoroughly washed with fresh distilled water and then, it was physically checked for any possible damage during surgical removal. This final cleaned skin was properly wrapped in a sterile aluminum foil, and carefully stored at -21°C and was used within a week.

### *Ex vivo* skin permeation studies

The Franz diffusion cells with 1 cm^2^ area and 10 mL volume of receiver cell were used to perform the *ex vivo* skin permeation studies on the skin of the rat. Full-thickness skin was mounted after removal of hair and fat on diffusion cells with water jacket to measure the permeability to the skin. The stratum corneum side of the skin was placed to face upward towards the donor compartment and dermal side was placed to face downwards to the receptor compartment. The donor cell was filled with 2 mL of OLE-NLC dispersion containing (1.5% OLE) and receptor medium was filled with permeation media at 37°C with 600 rpm. One mL of aliquot was collected from the receiver cell at specific time intervals for 24 h and an equal volume of the fresh receptor medium was filled immediately. The collected samples were diluted and were filtered using 0.45 mm membrane filter and analyzed using HPTLC (CAMAG, Switzerland). Same experiments were carried out for control formulation of UA for the comparison the permeation enhancement. The degree of permeation enhancement, enhancement ratio (ER) was determined as per the below given formula:

ER=(Steadystatefluxofformulation)/(Steadystatefluxofcontrol)

### *In vitro* release studies

The OLE- NLC formulation was converted into gel based on the highest entrapment efficiency and *ex vivo* transport data. The OLE-NLC gel was prepared by adding Carbopol-934 (1%, w/w) and set aside for the whole night for entire humectation of polymer chains. Some other components, e.g. 15% (w/v) PEG-400 and Triethanolamine (TEA) (0.5%, w/v) were also added to obtain the homogeneous dispersion of gel [32–33] and were taken for the assessment of analgesic and anti-arthritic activity. The control gel formulation (hydroethanolic solution 7:3) was also converted in gel as per the above procedure. The *in vitro* release experiments were performed for OLE- NLC gel formulations by paddle method. The release was performed with phosphate buffer (pH 5.5) and temperature was kept maintained at 32 ± 0.5°C to pretend both, pH and temperature of the human skin. Both the formulations were precisely weighed and putted in a dialysis bag which was closed from both sides. The whole assembly was kept at the base of the USP dissolution tester (VDA-8DR; Veego Scientific, Mumbai, India). The vessel contained buffer solution (500 mL) and speed was fixed to 50 rpm [34]. Aliquots (5 mL) were taken out from the release medium at different intervals of time and substituted by an equal volume of release medium. The released amount of the drug from the gel was found out by HPTLC. The obtained data from the undertaken release studies were then kinetically analyzed and the drug release pattern from both formulations were calculated.

## Pharmacological evaluation

### Animals

The healthy Wistar albino rats (100–150 gm) of both sexes were utilized for the *in vivo* study to assess the anti-arthritic activity of OLE-loaded NLC gel and marketed control gel formulation. All animals used in this study were acclimatized for one week by providing standard laboratory conditions. The animal rooms were maintained at 24 ± 1°C temperature; 45–55% relative humidity with 12:12 hours dark and light cycle. The animals were supplied with standard marketed pellet diet and distilled water *ad libitum*. The institutional guidelines on use and care of experimental animals were observed throughout the experimental work on the animals. The study protocol was approved by the Research Ethics Committee at HIT department, Jeddah Community College, King Abdulaziz University, Kingdom of Saudi Arabia (approval number: HIT/JCC-111-1438).

### *In vitro* anti-arthritic activity

In this study, the *in vitro* anti-arthritic activity of OLE- NLC gel and marketed gel formulation containing diclofenac sodium as standard were assessed by protein denaturation method. The denaturation mechanism, possibly involves modification in electrostatic hydrogen and disulphide bonding [35–36]. The reaction mixture (0.5 mL) contained 0.45 mL of bovine serum albumin (5% aqueous solution) and 0.5 gm each of both formulations. The pH was adjusted to 6.3 by using a little quantity of 1N HCl. The samples were then incubated at 37°C for 20 minutes and heated at a temperature of 57°C for the duration of 30 minutes. The sample was cooled and 2.5 mL of PBS (pH 6.3) was added in each sample. Similarly, the control test was performed and turbidity was precisely measured by the spectrophotometer at the wavelength of 660 nm. The percentage inhibition of the protein denaturation was then simply calculated by using the formula as given below.

%Inhibition={100−(ODofTest−ODofControl)X100}/(ODofControl)

### *In vitro* COX-1 and COX-2 assay

OLE-NLC gel and marketed gel formulation containing diclofenac sodium were evaluated for COX-1 and COX-2 inhibition. The COX inhibition screening standard assay kit (Catalog No. 760111, 760700; Cayman Chemical, USA) was used to perform the inhibitory assay of COX-1 and COX-2. The instructions supplied by manufacturer were followed [2].

## *In silico* Molecular Docking Analysis

To further strengthen the results of our *in vitro* and *in vivo* studies, we also performed *in silico* molecular interaction analysis on Ursolic acid and COX proteins using the Auto dock tool.

### Protein setup

Due to unavailability of crystal structural of rat COX enzymes, we retrieved the crystal structure of Ovis aries COX-1 (PDB ID: 1CQE) and Mus musculus COX-2 (PDB ID: 6COX) which is having more than 90% sequence homology rat Cox enzymes obtained from the Protein Data Bank available online [www.rcsb.org] [37]. The crystal structures PDB files were manually edited by removing heteroatom’s and only chain A of COX-1 and COX-2 were selected for docking studies. The protein structure’s quality was further improved by energy minimization using steepest descent algorithm, in GROMACS tool [38]. During structure minimization, the hydrogen were flexible and amino acid atoms were fixed at their coordinates. After reaching the saturated gradient, the complex structure was saved for further annotations. Finally the protein prepare for docking by adding charges (gigester), H-bonds (polar only) and neutralizing the histidine charges [39].

### Drug preparation and docking method

The drug Ursolic acid (CID 64945) co-ordinate was downloaded from the online PUBCHEM database [https://pubchem.ncbi.nlm.nih.gov/] [40] and atomic partial charges were then added to drug using PRODRG server [41], Audock 4.0 [42] tool used to perform molecular docking analysis in between protein and drug. Ligand parameter sets for docking analysis, as gyratory bonds kept flexible in docking with rigid protein structure. The blind docking were performed on rigid protein structure by giving the grid box dimension 70X60X60 at 0.375 Å center spacing. By using autorid4.0 the affinity maps for all the atoms (HD, C, NA, N, A, SA, Cl, S, Br, F, P and I) were calculated. Finally the Lamarckian genetic algorithm (LGA) search parameter was stimulated to activate the ligand-protein with one hundred simulations runs, with three hundred population size. The final docking results were prioritized based on the binding energies (lowest value).

### Docking analysis

Similar to experimental results, *in silico* molecular docking study shows that Ursolic acid binding strongly to COX enzymes by releasing free energy of (Cox-1) -10.3 K.Cal/Mol, (Cox-2) 8.9 K.Cal/Mol. The Cox-1 enzyme biding strongly with ursolic acid by forming four H-bond interaction (1.5Å) with four amino acids (Arg97, Gle347, Gln350 and Tyr355) of Cox-1. The Cox-2 amino acids (Thr212, Asn382) forming strong H-bond interactions with ursolic acid.

### Complete Freund's Adjuvant (CFA) induced arthritis in rats

The *in vivo* study in rats was undertaken to assess the anti-arthritic activity of OLE-loaded NLC gel and control gel formulation (OLE free NLC gel). The rats were picked randomly and divided into four experimental study groups. There were six animals in every experimental study group. Group-I (Normal control): treated with OLE free NLC gel (applied topically); Group-II (CFA. Toxic control): No drug treatment; Group-III (Treated group): Treated with OLE loaded NLC gel (1.2% applied topically); Group-IV (Positive control): Treated with marketed gel containing 1.16% diclofenac applied topically. The study was carried out for 21 days. On day 0, all the groups except group-I were inoculated by intradermal injection of CFA. The CFA suspension (0.1mL) was injected into the sub-plantar area of the left hind paw of all animals in each group. The CFA mainly contains the suspension of heat-killed *Mycobacterium tuberculosis* (1 mg/mL) into mineral oil. CFA causes inflammatory responses within 24 hours after injection. The volume of the hind paw was estimated by using the digital Plethysmometer. The paw volume was taken as parameter to measure the anti-arthritic activity of newly prepared NLCs and standard control marketed diclofenac gel [43].

### Biochemical estimation and radiological analysis

For the analysis of biochemical parameters, the blood sample was collected from all groups of rats into anticoagulation tubes. All required reagents were precisely added to find out the Erythrocytes (Red blood cells) count, Leucocytes (White blood cells) count [44], Erythrocyte sedimentation rate (ESR) [45], and Hemoglobin (Hb) contents [1]. Radiological analysis was also done to confirm the activity. The images were taken and safely compared.

### Inflammatory mediators analysis

The cytokines inflammatory mediators e.g. tumor necrosis factor alpha (TNF-α) and Interleukin-1 (IL-1) were precisely estimated using the ELISA kits methods (Biosource Int., Camarillo, CA, USA) as per the information supplied by the manufacturer of the kits [2].

### Analgesic activity

Analgesic activity of the test samples was performed by the hot water immersion tail-flick method. The treatment was given by gently applying the formulations of each group on the dorsal surface of the paw with the index finger. Five-centimetre portion of the tail of animals was immersed into hot water at 55 ± 5°C after 15 minutes treatment of gel. It was observed that in response to thermal stimuli, rats were quickly responded to withdrew their tail within few seconds. The cut off time (50 seconds) was undertaken to avoid any physical damage to the tissues of the tail of the rats. Time at which the animals withdrew their tail was noted [46].

## Statistical analysis

Statistical analysis of the data was done by analysis of variance (ANOVA) using Prism software (Graph Pad, version 5.0 San Diego, CA, USA). All values in this experiment are articulated as mean ± SEM. The values were regarded as significant, if P value was found p<0.05.

## Results and discussion

### Preparation and determination of Ursolic acid content of the extract

**Fig 2:**
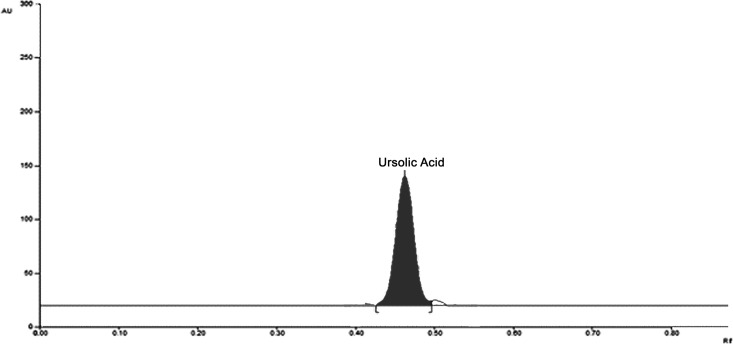
HPTLC chromatogram showing ursolic acid peak (R*f* = 0.49).

Preparation of OLE was done by adopting the method explained previously in the methods section. The extract was prepared with the yield of 8.85 ± 0.97% w/w in comparison with the weight of dried powder. The extract powder was found dark brown in color. UA was qualitatively estimated in extract of *O*. *sanctum* by HPTLC. A sharp peak was found at Rf value 0.48 (Fig 2). Based on HPTLC analysis, UA was revealed as the key constituent in the extract. The extraction method was efficiently increasing the UA content in the extract to 60.81 ± 5.23% w/w.

### Preparation and characterization of OLE loaded NLC

A total of ten NLC formulations were prepared by varying the ratio of solid lipid, liquid lipid, and surfactant, keeping drug concentration constant. The prepared formulations were characterized for particle size, PDI and TEM etc.

### Analysis of particle size and zeta potential

**Fig 3:**
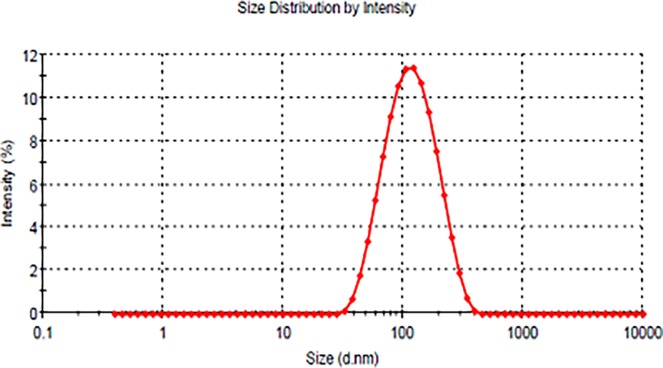
Size distribution of OLE-NLC.

The obtained results are given in the Table 1 (Fig 3). The mean particle size of OLE-free NLC and OLC-loaded NLC was found to be smaller than 130 nm with the PDI values of lesser than 0.2 demonstrating that the system has moderately narrow size distribution.

**Table 1:** Particle size, polydispersity index (PI) and zeta potential values of OLE-loaded NLC and OLE-free NLC.

Formulations	Particle size Mean diameter (nm)	Polydispersity index (PDI)	Zeta Potential (mV)
OLE-loaded NLC	120 ± 5.8	0.162 ± 0.022	-27.6 ± 3.5
OLE-free NLC	107± 8.7	0.132 ± 0.016	-25.5 ± 2.7

### Transmission electron microscopy (TEM)

**Fig 4:**
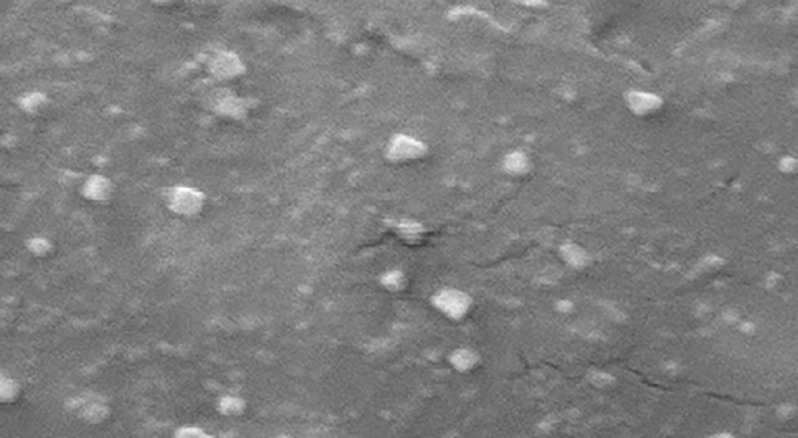
Transmission electron microphraph (TEM).

In order to get some additional information on the morphology and particle size, TEM was also done to take photos of OLE-NLC (Fig 4). As anticipated, particles found to be a spherical shape with varying size ranging approximately from 90 to 120 nm. Compared to diameter determined by DLS, the diameter observed by TEM was smaller, which might be explained by saying that the two methods were depending on different batches of samples and diverse principles [26,47]. It was reported that diverse morphologies of NLC were observed by different microscopes [48–49]. These discrepancies might be resulted due to lipid compositions and surfactants and/or due to the photographic tools used [50].

### Entrapment efficacy (EE)

The entrapment efficiency (EE) is an important physicochemical characteristic of lipid nano-particles as carriers of drugs. It is defined as their capacity for drug encapsulation. Based on the literature, a number of drugs have been effectively trapped in the SLN and NLC [51]. In comparison of the encapsulation efficiency between SLN and NLC, it was revealed that NLC showed a higher capacity of drug loading and entrapment efficiency (E.E.) [52–53]. In addition to this, it was also examined that the amount of encapsulated drug enhanced on raising the amounts of oil [29], which attributed to the better solubilization of lipophilic drugs in oils than in solid lipids. In the present investigation, EE of the best formulation was 89.56 ± 6.58%.

### *Ex vivo* skin permeation studies

The *ex vivo* transport study profile of all the formulations showed enhanced skin permeation data in comparison to control formulation. The maximum transdermal flux value was 92.51±11.47 μg/cm^2^/h over control formulation (34.39 ±12.54 μg/cm^2^/h) with an improved ratio of 2.69 across rat's skin. The developed formulation exhibited biphasic kinetics, with an elevated initial flux of UA during the first 3 hours followed by a decreased flux over the 3–12 hours period of time. These results printed out that the type of oil and surfactant display vital role in UA permeation in the course of rat's skin. On a therapeutic Consideration, a high initial flux can be regarded as beneficial since adequate quantity of biologically active ingredients are quickly released from the gel to the skin to exhibited an primary therapeutic action followed by a desired controlled release of the remaining bioactive components from the NLC. The flux and permeation of the drug through the rat skin were calculated using the following two equations. Equation-I is *J* = *Q*/(*A X t*) Where J is flux, Q is the quantity of compound traversing the membrane in time t, and A is the area of exposed membrane in cm^2^ and Equation-II is Kp = J/C_0;_ Where J is flux, C_0_ is initial concentration of drug and Kp is the permeability constant.

### *In vitro* release evaluations

The *in vitro* release evaluations on both conventional OLE gel and OLE loaded NLC gel demonstrated a prolong released characteristics. The developed formulation exhibited a biphasic pattern with a surge release for the duration of the first 3 hours, followed by an extended release maximum (82%) up to 12 hours. Conversely, a rapid release rate (96%) of the UA from conventional OLE gel was found as compared to OLE loaded NLC gel. It might be due to the diverse melting characteristics between liquid lipid and solid lipid. The solid lipid having a higher melting point may possibly crystallize first, which results small or no liquid lipid trapped into the core throughout the process of cooling. Lastly, many liquid lipids were situated on the superficial surface of a shell of the nano-particles which could be credited to fast drug release at the initial stage detected above [29]. Therefore, the rupture of nano-particles demonstrated the surge release at the beginning stage and subsequently sustained extended release.

## Pharmacological evaluation

### *In vitro* anti-arthritic activity of OLE-NLC-gel formulation

This is well documented and established fact that denaturation of protein is an important and major manifestations of arthritis. Formation of auto-antigens as in the case of rheumatoid arthritis might be due to de-naturation of proteins. Diclofenac gel (1.16%) was taken as a standard gel to compare the results with developed NLC gel formulation. The results revealed that standard gel showed percentage inhibition of 87.45%, whereas OLE-NLC showed percent inhibition of 84.67%. The above results showed that our laboratory formulation efficiency was comparable with that of the standard gel.

### Analgesic activity OLE-NLC-gel formulation

The results of analgesic activity in rats showed that the thermal stimulus time (TST) in animals of the negative control group was found to be 15.43 seconds, whereas standard gel formulation showed TST of 28.85 seconds. OLE-loaded NLC gel group animals showed TST as 34.87 seconds which is higher than standard (p<0.01). The control formulation showed TST of only 19.88 seconds which is higher than that of negative control. The enhancement in analgesic activity in case of developed formulation is due to higher penetration of drug into skin as compared to control and standard treatment. This is evident from the above results that OLE-NLC-gel formulation exhibited better analgesic activity as compared with the standard control gel.

### Effect of OLE-NLC-gel formulation on volume of Rat's paw in CFA induced arthritis

The Paw volume of the rats was recorded on 0, 5th, 12th and 21st day from the initiation of the study. The results indicate that there was an increment in paw volume of all groups from Day 0–5. OLE-NLC-gel formulation showed comparable results to those of the standard marketed gel formulation which supports the therapeutic potential of the test formulation (Table 2). Whereas, toxic group showed continuous increase in paw volume due to no-treatment. The CFA induced chronic inflammation is comparable to clinical situations of arthritis. The redness and swelling was shown over 24 h periods in the injected foot with irritation in the arthritis caused by CFA. The inflammatory reactions provoked by CFA induced the arthritis in a few days [54]. CFA increases the migration of monocytes/macrophage and lymphocytes into the synovial cavity of the affected area by inflammation.

**Table 2:** Effect of application of OLE loaded NLC on paw edema in rats.

Treatment	Paw volume (mL)			
	0 Day	5 Day	12 Day	21 Day
**Group-I: OLE-FREE-NLC-Gel (Normal control)**	1.34 ± 0.08	1.30 ± 0.04	1.32 ± 0.05	1.31 ± 0.04
**Group-I I: CFA (Toxic control)**	1.9 ± 0.04	2.1 5± 0.05#	2.60 ± 0.08#	2.72 ± 0.02#
**Group-III: CFA+OLE-NLC -Gel**	1.4 ± 0.06	1.68 ± 0.07*	1.47 ± 0.06**	1.43 ± 0.07**
**Group-IV: CFA + Marketed Gel** **(1.16% diclofenac)**	1.4 6± 0.05	1.60 ± 0.03**	1.44 ± 0.02**	1.38 ± 0.03**

Values expressed as mean ± standard error of mean.

#p<0.01 as compared to control group

*p<0.05

**p<0.01 as compared to toxic control.

The comparisons were made by ANOVA followed by Dunnett’s test.

The arthritis induced by CFA found to develop into two types of reactions. The primary and secondary reactions. The swelling in hind paw was developed in primary reaction, while the swelling(inflammation) in front paw and nodules in tails and ear was the result of the secondary reaction. The OLE-NLC-gel formulation found to significantly (P<0.01) inhibit the immune response in CFA induced arthritic rats. This is due to the capability of OLE-NLC-gel formulation to control the acute inflammation by inhibiting the inflammatory mediator such as PGE2 and reducing the vascular permeability (Table 2)

### Effect of OLE-NLC-gel formulation on hematological changes in rats of CFA induced arthritis

Table 3 displayed the CFA induced the hematological disturbance like reduced level of erythrocytes and hemoglobin (Hb) and increased level of leucocytes (WBC), erythrocytes sedimentation rate (ESR) in comparison with the normal negative control group. These parameters were found significantly altered by OLE-NLC-gel formulation treatment. The anemia is a main problem during arthritic condition which is due to reduction in RBC count and decreased level of Hb. The prominent causes of anemia include abnormal storage of iron in synovial tissue, reticulo-endothelial system, increased leukocyte count and the bone marrow failure to produce more red blood cells in response to the anemia. There was a reduction in RBC count, decreased level of Hb and increased WBC count and increased ESR level in the CFA induced arthritic rats which showed the anemic condition. Treatment of CFA induced arthritic rats with OLE-NLC-gel formulation was found to significant (P<0.01) decrease the leukocyte count. The mechanism of arthritic effects of OLE-NLC-gel formulation might be due to the immune system stimulation and might have the immunomodulatory effect, ESR-(Table 3) [1].

**Table 3:** Effect of OLE loaded NLC on hematological, inflammatory mediators and cyclooxygenase enzymes.

Treatment	Hematological parameters				Inflammatory mediators		COX Inhibition (%)	
	RBC (million/cu.mm)	WBC (cells/cu.mm)	ESR (mm/h)	Hb (gm%)	TNF-α (ng/mL)	IL-1 (ng/mL)	COX-1	COX-2
**Group-I: OLE-FREE-NLC-Gel** **(Normal control)**	7.11 ± 0.83	6.6 ± 0.65	2.4 ± 0.31	13.4 ± 1.50	0.13 ±0.05	0.38 ± 0.07	---	---
**Group-I I: CFA (Toxic control)**	4.13 ± 0.06#	9.81± 1.2#	3.2 ± 0.52#	9.8 ± 0.81#	0.45 ±0.10#	0.0.98±0.06#	---	---
**Group-III: CFA+OLE-NLC -Gel**	6.74 ± 0.08*	7.12 ± 0.05**	2.7 ± 0.20 **	12.5± 1,2**	0.16 ±0.06	0.44± 0.05**	48± 5.76	60±10.42
**Group-IV: CFA + Marketed Gel** **(1.16% diclofenac)**	6.40 ± 0.04**	7.5 ± 0.03	2.6 ± 0.41**	11.9± 0,87**	0.14 ±0.03**	0.42 ± 0.08*	57 ± 8.4	50 ± 8.87

Values expressed as mean ± standard error of mean.

#p<0.01 as compared to control group

*p<0.05

**p<0.01 as compared to toxic control.

The comparisons were made by ANOVA followed by Dunnett’s test

### Effect of OLE-NLC-gel formulation on pro-inflammatory mediators in rats of CFA induced arthritis

The results of the treatment of OLE-NLC-gel formulation to CFA induced arthritic rats showed significant inhibition to the pro-inflammatory cytokines like IL-1 and TNF–α as shown in the Table 3. The outcome of these results indicated that the anti-inflammatory potential of OLE-NLC-gel formulation against the CFA induced inflammatory condition, which is very similar to clinical inflammatory manifestations. Since OLE-NLC-gel formulation showed the anti-inflammatory effect against CFA induced arthritis, then further, it was considered worthwhile to evaluate the effect of the OLE-NLC-gel formulation against COX-2 and COX-1 to further elucidate the potential mechanism of anti-inflammatory action (Table 3). The level of pro-inflammatory cytokines like IL-1 and TNF-α in the knee joint and serum was found to be increased in the RA patients [55]. Numerous inflammatory biomarkers and pro-inflammatory molecules are reported to investigate the inflammation during the arthritis. There is a correlation of increased serum level of cytokines and Arthritis.

There are various the inflammatory mediators which play vital role in the progress of the inflammation in the joint. A variety of cytokines e.g. interleukins (IL-1, IL-6, IL-8-6, IL-12 etc.) and TNF-α are reported to be involved in the pathogenesis of RA. IL-1 and TNF- α collectively responsible to engage the leukocytes into the inflammatory joint in arthritis. TNF-α is a pleiotropic cytokine, mainly secreted by activated macrophages and monocytes. TNF-α regulates the immune cells and plays a vital role in both chronic and acute inflammation. TNF promotes the inflammatory response which causes autoimmune disorders like ankylosing spondylitis, rheumatoid arthritis, and inflammatory bowel disease, etc. TNF-α is reported to be found in the serum and synovial fluid of arthritic patients and it has been linked with clinical symptoms as well as a laboratory marker of RA. The main approach to inhibit the progress of the RA by blocking the TNF-α signal by two ways. Firstly, to decrease the severity of inflammatory reactions and secondly by decreasing the damage to the bone in CFA induced arthritis in rats. IL-1 is an important inflammatory mediator which destroyed the synovial cells by promoting the prostaglandin (PGE2) synthesis and by production of fibroblast in the synovial fluid [56–57]. Our results revealed that OLE-NLC-gel formulation possess a significant anti-arthritic effect as shown by the inhibition of above pro-inflammatory mediators.

### *In silico* Molecular Docking Analysis

**Fig 5:**
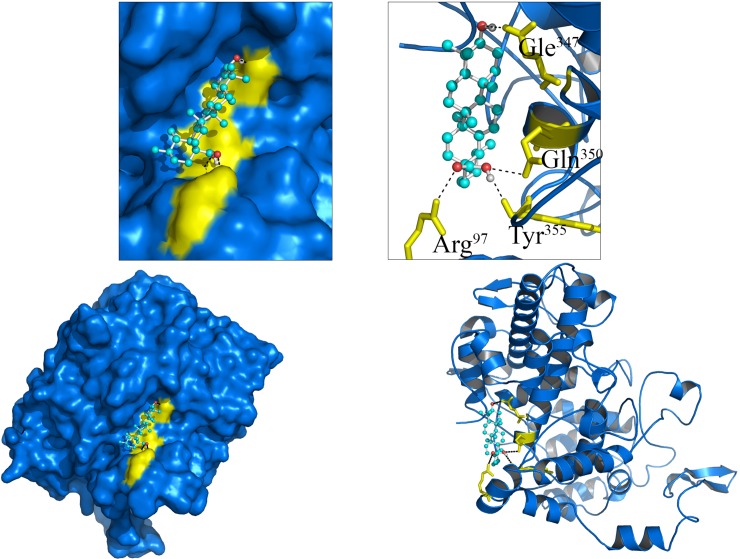
Molecular interactions of Ursolic acid (Ball and Stick) with Cox-1(Blue Cartoon/Surface).

**Fig 6:**
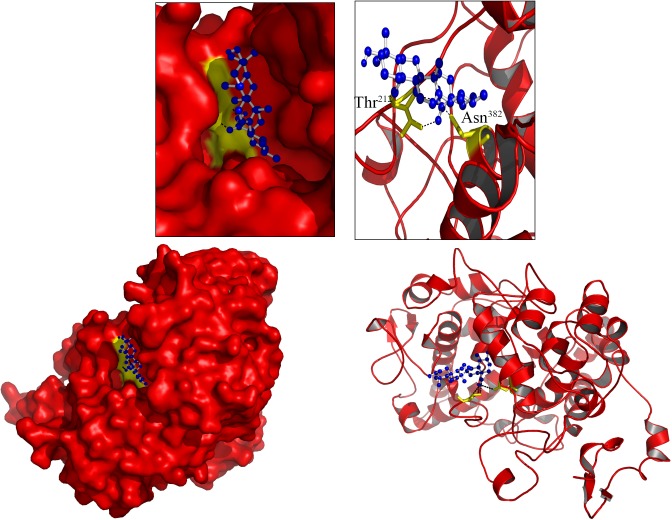
Molecular interactions of *Ursolic acid* (Blue ball and stick) with Cox-2 (Red Cartoon/Surface).

Similar to experimental results, *in silico* molecular docking study shows that Ursolic acid binding strongly with COX enzymes by releasing free energy of (Cox-1) -10.3 K.Cal/Mol, (Cox-2) 8.9 K.Cal/Mol (Fig 5). The Cox-1 enzyme biding strongly with ursolic acid by forming four H-bond interaction (1.5Å) with four amino acids (Arg97, Gle347, Gln350 and Tyr355) of Cox-1. The Cox-2 amino acids (Thr212, Asn382) forming strong H-bond interactions with ursolic acid (Fig 6).

### Radiological studies

**Fig 7:**
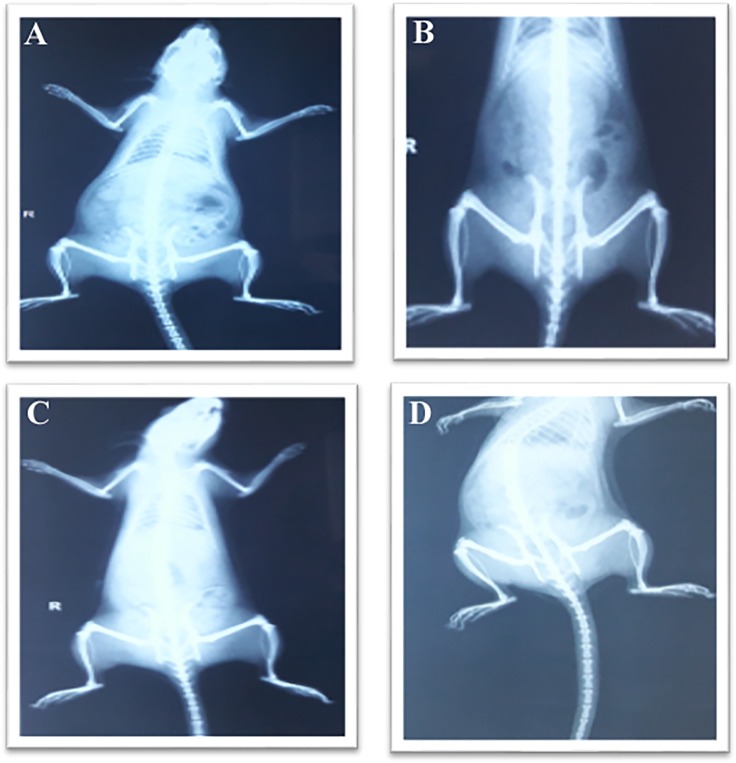
Effect of OLE loaded NLC gel in CFA induced arthritic rats (X-ray photographs). A: Group-I. Normal control; B: Group-II. Toxic control; C: Group-III. OLE loaded NLC gel; D: Group-IV. Marketed gel.

X-rays of the hind paw of all groups of rats were taken for the evaluation of the bone damage (Fig 7). In Fig 7(**A**), the radio graphical analysis indicates no arthritic condition. In Fig 7(**B**), This group did not receive any treatment, so severe arthritic condition was seen in this group. In Fig 7(**C**) and 7(**D**), the X-ray of rat showed good recovery from arthritis, which was treated with OLE-NLC-gel formulation and standard diclofenac gel respectively. The good recovery in the given state might be attributed to the better therapeutic efficacy of OLE-NLC-gel formulation in arthritis and due to greater augmented penetration of OLE-NLC-gel formulation into skin.

## Conclusions

The preparation of OLE-loaded NLCs were successfully done by adopting a solvent evaporation method and investigated for *in-vitro* and *in vivo* anti-arthritic activity. The gelling agent known as carbopol-934 was suitably utilized to convert the optimized NLCs formulation into gel. In the present study, it was found that OLE-loaded NLC provides enhanced transdermal flux value across rat skin. The in vivo data revealed that the analgesic effect of OLE-NLC gel was comparable to that of standard gel. Rat paw volume showed a depriving effect with enhanced RBC count and decreased WBC count. The results obtained in COX-1 and COX-2 study indicated that developed formulation has inhibitory effects against these enzymes, and also reduced the IL-1 and TNF-α levels. The above observations make it evident our in-house OLE- NLC gel was effective in arthritis management. Further, corroboration was done by radio-graphical analysis, which depicted a good recovery phase in the group treated with developed formulation.
